# The Influence of Flame Retardants on Combustion of Glass Fiber-Reinforced Epoxy Resin

**DOI:** 10.3390/polym14163379

**Published:** 2022-08-18

**Authors:** Oleg Korobeinichev, Artem Shaklein, Stanislav Trubachev, Alexander Karpov, Alexander Paletsky, Anatoliy Chernov, Egor Sosnin, Andrey Shmakov

**Affiliations:** 1 Institute of Chemical Kinetics and Combustion, 630090 Novosibirsk, Russia; 2 Udmurt Federal Research Center, 426067 Izhevsk, Russia; 3 Department of Physics, Novosibirsk State University, 630090 Novosibirsk, Russia

**Keywords:** flame spread, opposed flow, polymer composites, flame retardants, numerical modeling, temperature measurement, glass fiber reinforcement, pyrolysis, flammability

## Abstract

For the first time, next to the flammability tests (LOI, UL-94 HB, VBB, TGA), experimental tests and computer simulation have been conducted on the flame spread and combustion of glass fiber-reinforced epoxy resins (GFRER) with 6% graphene and 6% DDM-DOPO flame-retardant additives. The downward rates of flame spread (ROS) in opposed flow with oxidizer and the upward ROS along GFRER composites have been first measured as well as the distribution of temperature over the combustion surface of the composites with flame-retardant additives and without them. The LOI and UL-94 HB tests showed a reduction in the flammability of GFRER when flame retardants were added and predicted a higher effectiveness of DDM-DOPO compared to graphene. Adding DDM-DOPO resulted in increasing the rate of formation of the volatile pyrolysis products and their yield, indicating, together with the other data obtained, the gas phase mechanism of the flame retardant’s action. Adding graphene resulted in an increase in the soot release on the burning surface and an increase in the amount of non-volatile pyrolysis products on the burning surface, reducing the amount of fuel that participated in the oxidation reactions in the gas phase. The developed numerical combustion model for GFRER with a DDM-DOPO additive, based on the action of DDM-DOPO as a flame retardant acting in the gas phase, satisfactorily predicts the effect of this flame retardant on the reduction in downward ROS over GFRER for 45–50% oxygen concentrations. The developed model for GFRER with graphene additive, based on a reduction in the amount of fuel and increase in the amount of incombustible volatile pyrolysis products when graphene is added, predicts with good accuracy downward ROS over GFRER depending on oxygen concentration.

## 1. Introduction

Glass fiber-reinforced plastics based on epoxy resins are some of the most promising structural polymer materials. These materials are widely used in different industries, primarily in the aircraft industry. Next to durability, the non-combustibility of structural materials is their important characteristic. Reinforcement of the material with glass fiber reduces its combustibility a little and improves its mechanical properties, but the binder (epoxy resin) remains easily combustible. During the thermal decomposition of epoxy resin, char is formed, which may protect the unburnt polymer from the heat generated by flame; however, the protective properties of char are insufficient for the material to pass a stringent industrial standard flammability test, such as UL-94 V0. Thus, the addition of flame retardants is one of the practical means to improve the flame retardancy of an epoxy resin system [1,2]. Recently, the highly efficient flame-retarded epoxy resin systems based on the diglycidyl ether of bisphenol A (DGEBA) resin have been presented [3,4,5,6]. Flame retardant may affect the thermal decomposition of a polymer as well as inhibit the oxidation of volatile gaseous products [7].

Over the many years of research of the combustion of various polymer materials, a large number of flame retardants have been developed. Several decades ago, flame retardants for polymer materials were mostly halogen-containing substances (for the most part, based on bromine and chlorine); however, adding them significantly increased the toxicity of the products of pyrolysis and combustion of the material. In [8], brominated dioxins and furans from the pyrolysis of a brominated diphenyl oxide were detected at 510–630 °C. Therefore, a large number of studies began to be published, which were aimed at the synthesis of flame retardants not containing halogens [9]. At the time of this writing, there exist flame-retardant additives based on phosphorus, nitrogen, graphene and different metals. Yet, flame retardants based on phosphorus such as ammonium polyphosphate [10], 9,10-dihydro-9-oxa-10-phosphaphenanthrene-10-oxide (DOPO) [11,12] and graphene oxide [13] proved to be most common.

Phosphorus-containing flame retardants may reduce the flammability of a polymer both by forming a carbonaceous frame (char) in the condensed phase and by inhibiting gas-phase reactions. There are three mechanisms by which the formation of char reduces the combustibility of solid fuel: (1) part of the carbon (and hydrogen) remains in the condensed phase, thus reducing the yield of gaseous products upon decomposition; (2) the char layer of low thermal conductivity serves as a thermal insulator for the polymer, and (3) the dense char acts as a physical barrier for the yield of gaseous fuel degradation products [14]. In the gas phase, phosphorus-containing flame retardants accelerate the recombination of H and OH radicals, thus slowing down the combustion reaction in the gas phase [15]. The combustibility of glass fiber-reinforced composites and the impact of different additives on their combustion were actively investigated in [16,17,18]. In [19,20], the thermomechanical properties of fiber-reinforced composites at higher temperatures were studied. In these works, the thermal degradation and combustion of composites were studied in thermogravimetric tests, cone calorimeter tests, and the main thermophysical parameters of the samples were measured, as the thermal conductivity of solid fuel has a great impact on the flame spread. In the works by Kandare et al., the influence of certain flame-retardant additives (cellulose, melamine phosphate) on the flammability and mechanical properties of fiber-reinforced epoxy composites was examined. Using TGA, the kinetic parameters of thermal degradation of glass fiber-reinforced composites were determined.

As a rule, the influence of flame retardants on the combustion of glass fiber-reinforced composites was investigated on the basis of TG tests, the cone calorimeter test, UL-94, LOI, SEM (scanning electron microscopy), and FTIR [10,21,22,23]. Certain authors indicate the presence of synergism at the simultaneous action of graphene and phosphorus-containing flame retardants in composite materials based on epoxy resins [24,25]. However, flammability tests do not always provide precise information on the behavior of a material under conditions of a fire. In this case, a more precise prediction of the behavior of materials and the mechanism of the action of flame retardants may be obtained on the basis of experimental data on the flame spread over a polymer. For instance, the limits of flammability of fire-proof materials (meta- and para-aramid fabrics (NOMEX and Kevlar), poly-imide film (Kapton) and polycarbonate (PC)) in the opposed oxidizer flow were investigated in [26]. Flame spread over carbon fiber-reinforced plastics (CFRP) in an oxidizer flow was investigated in [27,28]. The authors found that the high thermal conductivity of the carbon fiber controls flame spread over CFRP.

When investigating flame spread over fire-resistant solid fuels, in order to maintain their combustion, the authors [29] used an external heat flow onto the surface of the fuel, and the authors [26] raised oxygen concentration in the gas phase. Such studies allow researchers better to understand the mechanisms of fire emergence and growth and may be useful in developing more effective fire-resistant materials.

The general goal of this study was to investigate the mechanism of reducing the flammability of fiber-reinforced epoxy resin (GFRER) by adding graphene and DDM-DOPO flame-retardant additives.

The majority of the available works on the flammability of fiber-reinforced polymer materials with flame retardants are devoted to the study of the behavior of materials in different tests, and little attention is paid to the spread of flame over them, which is the case under conditions of a fire. In addition, there are no data in the literature on the development of models and numerical simulation of flame spread over glass fiber-reinforced plastics both without the addition of flame retardants and with additives. Thus, the novelty of this research is an experimental study of the mechanism of the flame-retardant effect on the combustion of glass fiber-reinforced epoxy resins not only under standard flammability test conditions but also under conditions of flame spread at an increased oxygen concentration and the presence of an external flame source. The novelty of this study consists in the development of a model and the numerical simulation of flame propagation over reinforced GFRER composites without the addition of flame retardants and with the addition of 6% graphene and 6% DDM-DOPO, which makes it possible to predict the downward flame spread over these materials. 

It should also be noted that the importance and relevance of this study lies in the need to reduce the flammability of fiber-reinforced epoxy resin, which is promising for use in the aircraft industry due to the danger of fires and deaths on board the aircraft.

## 2. Experimental Section

### 2.1. Materials

The research includes a number of studies on the flammability of glass fiber-reinforced polymers with 6% graphene and 9,10-dihydro-9-hydroxy-10-phosphaphenantrene-10-oxide-4,4′-diaminodiphenylmethane (DDM-DOPO) flame-retardant additive and without them. The sample slab’s thickness was 0.95 mm. The slabs were prepared from prepreg based on T-15 (P)-76 fabric (92) by vacuum forming. There were 6 layers in the glass fiber fabric. The binder content in the prepreg was 35%. The binder composition included resin diglycidyl ether of bisphenol A (DGEBA, CAS 1675-54-3), which was obtained from Sigma-Aldrich Co. (St. Louis, MO, USA). The use of this resin demonstrated better, compared with epoxy resin, effectiveness of reducing the combustibility of the composites [4]. The curing agent was 4,4-diaminodiphenylmethane (DDM) (CAS 101-77-9), which was obtained from Sigma-Aldrich Co. (USA). The curing agent DDM was introduced into the DGEBA resin as fine powder. Graphene was produced by the RUSGRAPHENE company. The photographs of the GFRER + 6% graphene surface are shown in Figure S1 (Supplemental Materials). DDM-DOPO was provided for the tests by Prof. Yuan Hu from the USTC China. The method of synthesizing DDM-DOPO was previously described in [12]. Before being introduced, graphene was dispersed in an ultrasonic bath in acetone in the ratio of 1 g graphene to 100 mL acetone during 1 h at room temperature. After that, epoxy resin was added to graphene dispersion in acetone and was stirred for 2 h. Then, the mixture was heated in an oil bath at the temperature of 130 °C during 0.5 h to remove acetone. After that, the mixture was cooled to room temperature, finely ground DDM powder was added to it, and the mixture was stirred for 2 h. Before DDM-DOPO and the curing agent (DDM) were introduced into epoxy resin, they were ground to a fine powder and then mixed with the resin during 2 h at room temperature. The obtained mixtures of resin, graphene and DDM, as well as of resin, DDM-DOPO and DDM, were used for making prepregs. The glass fiber matrix was oriented in one direction for all the layers of the prepreg, indicating the one-directional structure of the fiber reinforcement. The curing mode was as follows: 100 °C for 2 h, 150 °C for 2 h. The density of the obtained sample slabs was 1420, 1430 and 1480 kg/m^3^ (±30 kg/m^3^) for GFRER, GFRER + 6% DDM-DOPO and GFRER + 6% graphene, respectively.

### 2.2. Thermal Degradation Analysis

The thermal decomposition of the samples was studied using thermogravimetric analysis (TGA). Pieces of GFRER slabs weighing 3–4 mg were placed in an aluminum crucible using a synchronous TG/DSC analyzer STA 409 PC (Netzsch) in a 100 v% helium and 79 v% He + 21 v% O_2_ flow with a volumetric velocity of 27 cm^3^/min (NTP). The samples were heated from 30 to 580 °C at the heating rate of 30 K/min. All the experiments were repeated at least 3 times.

### 2.3. LOI, UL-94 HB

The LOI (in accordance with ISO 4589-2) and UL-94 HB (according to EN 60695-11-10) tests were performed for the GFRER composites under study with 6% graphene and 6% DDM-DOPO flame-retardant additives according to standard methods. The accuracy of determining LOI was ±0.1%.

### 2.4. Downward Flame Spread Experiments

The experimental setup used for the tests was similar to that used in [18]. The sample slabs of GFRER with 6% graphene or DDM-DOPO additive or without it were inserted into a thin aluminum frame (sample holder) 2 mm thick to prevent the flame spread along the side surfaces, while the width of the open surface of the sample (over which the flame propagated) was 20 mm. The sample length was 75 mm. The sample and the frame were marked with a step of 10 mm to measure the rate the flame spread (ROS) from the video recording of the experiments with a FujiFilm x-A20 camcorder (the shooting frequency was 30 frames per second).

**Figure 1:**
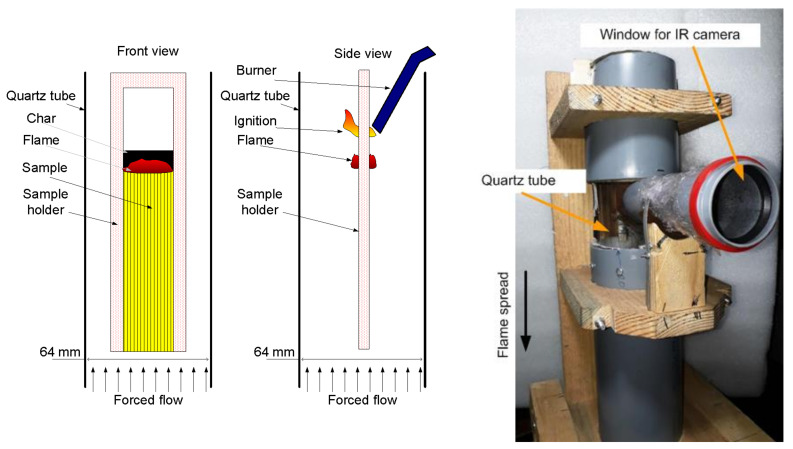
The schematic of the experimental setup (front view—(**left**), side view—(**center**)) and its photo (**right**).

The experimental setup for studying downward flame spread is shown in Figure 1. The sample was suspended in a cylindrical transparent quartz tube with a diameter of 64 mm and a length of 45 cm using a duralumin holder. Using MKS flow controllers, a mixture of N_2_ and O_2_ of various concentrations (30–50 v% O_2_) was fed into the tube through polyethylene hoses. A honeycomb, a foam rubber flow equalizer, was installed in the pipe at the inlet. For all types of samples and oxygen concentrations, the flow rate was fixed during the experiment at 4 cm/s. The sample was ignited from above using a propane–butane burner after turning on the opposed oxidizer flow (the process of ignition of the sample with the burner is schematically shown in Figure 1 (the center)). An opening was made in the pipe to which a short viewing pipe was glued that was 50 mm in diameter and covered at the end with polyethylene film 0.005 mm thick passing infrared radiation. Through the viewing pipe, the thermal image of the sample surface was recorded using an IR camera Guide C400, which was followed by the calculation of temperature on the sample surface as a function of time. The transmission factor of the polyethylene film and the sample surface radiation factor were determined by way of calibration with a Pt-PtRh10% thermocouple 50 micron thick embedded into the surface of the same sample. The recording frequency of the IR camera was 1 Hz.

When the GFRER sample was burnt, the combustion products entered the gas phase to form soot deposits on the surface of the burnt sample. After burning of the sample, soot was collected from its surface, and its weight was measured. The complete yield of the combustion products into the gas phase, including gaseous volatile products and soot deposits, was determined as the difference in mass between the original sample and the burnt sample, which was rated by the mass of the original sample.

### 2.5. Vertical Bunsen Burner Test

**Figure 2:**
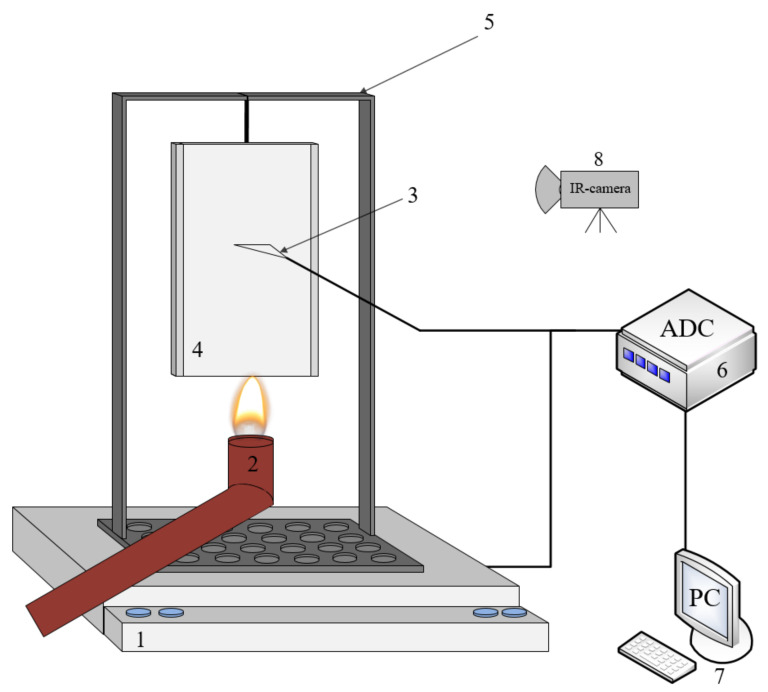
The experimental setup for VBB test. 1—electronic balance, 2—burner, 3—thermocouple for calibration, 4—sample, 5—sample holder, 6—ADC, 7—PC, 8—IR-camera.

The vertical Bunsen burner test (VBB) (ASTM D3801-20a) is used to assess flammability and the behavior of solid materials at burning. In the study, its configuration was used, which was similar to that used in the above standard. The experimental setup for the case was similar to that used in [30]. In the experiment, the sample with the dimensions of 30 × 80 mm^2^ was located vertically above the outlet of the burner, as shown in Figure 2. The aluminum holder covered the sides of the sample, and the exposed surface was 20 mm wide. The burner was a copper pipe with the internal diameter of 11 mm and wall thickness of 1.5 mm. The lower edge of the sample was placed symmetrically relative to the burner outlet at the elevation of 24 mm. A laminar diffusion flame fueled by propane gas was maintained during the entire test. The fuel flow rate was set to be 0.5 ± 0.05 cm^3^/s. The transient variation of the sample weight was recorded with an electronic balance, onto which a sample holder was installed. Ignition and burning of the sample were recorded with a video camera. The sample surface temperature was recorded similarly to the case described in 2.3, but there was no polyethylene film between the IR camera and the sample. The camera was calibrated, accordingly, without the film. Tests were repeated at least 3 times to specify the mass loss rate to reach the relative 20% accuracy.

## 3. Numerical

### Formulation

To predict downward flame spread over GFRER, as well as flame spread over the horizontal surface of GFRER, the following mathematical model was formulated, which takes into account coupled heat and mass transfer between gas phase flame and solid fuel, multicomponent reacting gas flow, gas phase combustion, heat transfer and pyrolysis in a solid material [18,31,32,33,34,35]:

(1) $$\frac{\partial \rho }{\partial t}+\frac{\partial \rho u_{j}}{\partial x_{j}}=0$$

(2) $$\rho \frac{\partial u_{i}}{\partial t}+\rho u_{j}\frac{\partial u_{i}}{\partial x_{j}}=-\frac{\partial p}{\partial x_{i}}+\frac{\partial }{\partial x_{j}}\mu \frac{\partial u_{i}}{\partial x_{j}}+\left(\rho_{a}-\rho \right)g_{i}$$

(3) $$\rho C\frac{\partial T}{\partial t}+\rho u_{j}C\frac{\partial T}{\partial x_{j}}=\frac{\partial }{\partial x_{j}}\lambda \frac{\partial T}{\partial x_{j}}+\rho WQ-\frac{\partial q_{j}^{r}}{\partial x_{j}}$$

(4) $$\rho \frac{\partial Y_{k}}{\partial t}+\rho u_{j}\frac{\partial Y_{k}}{\partial x_{j}}=\frac{\partial }{\partial x_{j}}\rho D\frac{\partial Y_{k}}{\partial x_{j}}+\nu_{k}\rho W$$

(5) ρ=p/RT

Here, $x_{i}=\left\{x,y\right\}$, $u_{i}=\left\{u,v\right\}$, $k=\left\{F,O,P\right\}$, $\nu_{k}=\left\{-1,-\nu_{O},1+\nu_{O}\right\}$, $g_{i}=\left\{g,0\right\}$.

A single-step mechanism employed here to predict gas phase combustion was expressed as

(6) $$F+\nu_{O}O+I\to \left(1+\nu_{O}\right)P+I$$

in which the reaction rate is expressed in an Arrhenius form

(7) $$W=kY_{F}Y_{O}\exp\left(-E/R_{0}T\right)$$

Solid material was a composite (fiber-reinforced plastic) made of a combustible binder (epoxy resin) reinforced with non-combustible glass fibers. The model was formulated to take into account these two components. The energy conservation equation of the solid material was expressed as:

(8) $$\rho_{s}C_{s}\frac{\partial T_{s}}{\partial t}=\frac{\partial }{\partial x_{j}}\lambda_{s}^{j}\frac{\partial T_{s}}{\partial x_{j}}+\eta_{b}^{0}\rho_{b}Q_{b}W_{b}$$

A reaction rate of pyrolysis reaction is given by

(9) $$W_{b}={\left(1-\alpha \right)}^{n}k_{b}\exp\left(-E_{b}/R_{0}T_{s}\right)$$

where the conversion degree varies from 0 to 1 and is defined as

(10) $$\frac{d\alpha }{dt}=W_{b}$$

The kinetic parameters used in Equation (9) were determined from the TGA data.

Density of the solid material was defined taking into account binder burnout as follows:

(11) $$\rho_{s}=\eta_{b}^{0}\left(1-\alpha \right)\rho_{b}+\left(1-\eta_{b}^{0}\right)\rho_{f}$$

The local mass burning rate (combustible volatiles gasification rate) at a burning surface was defined as

(12) $${\dot{m}}_{b}(x)=\eta_{b}^{0}\rho_{b}\int_{0}^{L_{s}(x)}W_{b}dy$$

The gas-phase mechanism of action of DOPO-based flame retardants has been confirmed by numerous studies [11,12,23,36,37]. The use of DOPO-based flame retardants results in the release of PO into the gas phase, which results in effective flame inhibition [38]. Thus, based on the analysis of the experimental data, a DDM-DOPO flame retardant is assumed to take effect primarily in the gas phase. The pre-exponential factor of the gas phase combustion reaction is reduced in the following way

(13) $$k_{g,DOPO}=\left(1-\psi Y_{DOPO}\right)k_{g},$$

where $Y_{DOPO}$ is the initial mass fraction of DDM-DOPO in the solid composite material, and ψ is the inhibition effect coefficient.

The effect of a graphene-based flame retardant was taken into account based on the following considerations. The experimental observations show intensive soot formation during flame spread over GFRER slabs inhibited by graphene-based flame retardant. This soot accumulates on the thermocouples, as well as on the burning surface, which results in the growth of a porous structure on a composite sample. We presume that a part of the total gaseous pyrolysates $\left(1-Y_{F,s}\right){\dot{m}}_{b}(x)$ is non-combustible gas, which is further converted into soot, while $Y_{F,s}{\dot{m}}_{b}(x)$ goes to the gaseous fuel. $Y_{F,s}$ represents the inhibition effect coefficient. It is to be noted that in the model according to Equation (4) $\left(1-Y_{F,s}\right)$, part of the local mass burning rate (${\dot{m}}_{b}(x)$) is supplied to P (products). The boundary conditions for the governing Equations (1)–(4) and (8) are generally accepted and are presented elsewhere [31,35].

The density of the binder is $\rho_{b}$ = 1165 kg/m^3^, and the density of the glass fibers is $\rho_{f}$ = 1670 kg/m^3^. The mass fraction of the binder is $\gamma_{b}^{0}$ = 0.35, which corresponds to the volume fraction $\eta_{b}^{0}=\frac{\gamma_{b}^{0}/\rho_{b}}{\left[\gamma_{b}^{0}/\rho_{b}+\left(1-\gamma_{b}^{0}\right)/\rho_{f}\right]}$ = 0.43; thus, the initial density of the composite material is $\rho_{s}=\eta_{b}^{0}\rho_{b}+\left(1-\eta_{b}^{0}\right)\rho_{f}$ = 1440 kg/m^3^, which varies according to Equation (11).

The thermal conductivity of GFRER in a direction normal to glass fibers is $\lambda_{\mathrm{GFRER}}^{y}$ = 0.25 W/m/K [18,19]. The thermal conductivity of GFRER in a direction along the fibers is set to $\lambda_{\mathrm{GFRER}}^{x}$= 0.20 W/m/K [18,39]. The specific heat capacity of GFRER at temperatures close to the temperature of the burning surface is $C_{s}$ = 1400 J/kg/K [18].

The thermal decomposition of epoxy resin produces high yields of low-molecular gaseous components (methane, carbon monoxide) [40]. Thus, kinetic parameters of the gas-phase combustion reaction were set to be similar to previous studies [31,32,33]: activation energy of E = 90 kJ/mol and pre-exponential factor of k = 10^10^ 1/s. The heat release of GFRER combustion was set to be Q = 25.5 MJ/kg, according to the measurements [41].

The various gaseous products released by epoxy resin decomposition [40] make it impossible to provide reasonable arguments for some value of the stoichiometric coefficient $\nu_{O}$ in Equation (4). Thus, $\nu_{O}$ was set to be 1.7 based on preliminary calculations aimed at reaching an agreement between the measured and predicted flame spread rate.

## 4. Results and Discussion

### 4.1. Results of the TGA, LOI, UL-94 HB and VBB Tests

**Figure 3:**
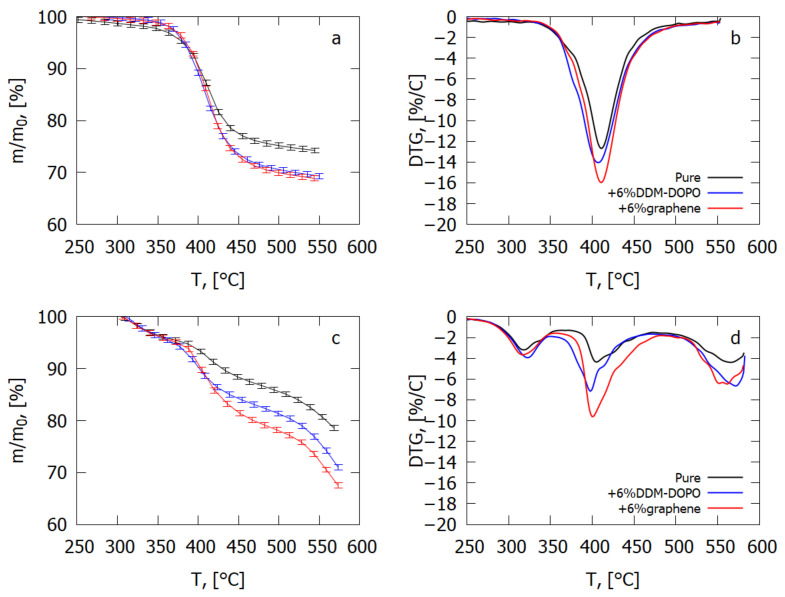
Thermogravimetric data of pure GFRER and GFRER composites. (**a**) TG in inert medium, (**b**) DTG in inert medium, (**c**) TG in air atmosphere and (**d**) DTG in air atmosphere.

Figure 3 shows a comparison of the thermogravimetric curves of the degradation of GFRER and GFRER with 6% graphene and 6% DDM-DOPO additives. The data for inert medium (He) are shown in Figure 3a,b, whereas the data for the «air» (He + O) medium are shown in Figure 3c,d.

One can see from Figure 3 and Table 1 that in inert medium, adding 6% graphene results in the increase in the thermal degradation velocity of the GFRER sample by 23% and in the increase in the total yield of the volatile pyrolysis products by 4–5%. Adding 6% DDM-DOPO results in a decrease in the temperature of the degradation rate maximum by 5 K and thus in an increase in the volatile products yield rate compared with the sample without additives. At the same time, the total yield of the volatile pyrolysis products increases by 4–5% due to a reduction in the char yield. In the case of GFRER without a flame-retardant additive, the char yield is 9%. In the case of composites with flame retardants, DDM-DOPO and graphene interact with the polymer in the condensed phase. When flame retardants are added, solid-phase reactions with the binder occur, leading to the formation of the additional amount of new volatile pyrolysis products, which are not formed during the decomposition of GFRER without additives. The release of these volatile products results in a decrease in the residue mass, which is visible on TG.

In air, the sample with 6% graphene additive degrades at a fast velocity and with a large yield of the volatile pyrolysis products compared with the sample with 6% DDM-DOPO additive. In addition, in oxidative medium, three phases of degradation were observed with the degradation rate maxima at ≈320, ≈400, and ≈560 °C. When flame retardants were added, no additional phases were observed; therefore, three degradation phases in air are related only to the binder. In [40], where the oxidative pyrolysis of epoxy resin based on DGEBA was investigated, it is stated that the oxidative destruction of epoxy resin cannot be unequivocally interpreted and includes the synergistic effects of pyrolysis proper and of heterogeneous oxidation. The authors of this study also relate that in air atmosphere, the initial gaseous products are formed at the temperature of around 300 °C, while large amounts of CO_2_ and CO are formed at a higher temperature. In the atmosphere of O_2_, new compounds of the oxygen group are formed upon the degradation of epoxy resin, which are absent at degradation in inert medium. The loss of the sample mass at the temperature of 450 °C (the second peak) is suppressed by the increase in the O_2_ concentration

In modeling of the flame spread over glass fiber-reinforced plastic, pyrolysis kinetics was used, which was obtained from TG and DTG in inert medium (Figure 3a,b), as the development of a model of thermal degradation depending on the share of oxygen near the sample surface is an essential complication. The major share of the yield of the pyrolysis products is observed in the combustion zone far from the flame front, where there is no oxygen near the glass fiber-reinforced polymer surface, and pyrolysis of the binder occurs practically in inert medium. Assuming that the pyrolysis reaction occurs in one stage and is of the first order, from the data in Figure 3a,b, the kinetic parameters of pyrolysis were obtained using the established method [42]. The obtained kinetic parameters for GFRER were: *n* = 1, E = 166 kJ/mol and A = 6.97 × 10^10^ 1/s. In the case of DDM-DOPO, the same kinetic parameters of pyrolysis were determined. In the case of graphene, the kinetic parameters obtained were: *n* = 3, E = 267 kJ/mol and A = 1.4 × 10^19^ 1/s. The accuracy of determining the pyrolysis rate constant by this method is factor 10.

The LOI and UL94 data, the mean mass loss rate (MLR), and the total mass loss (TML) in the VBB test are shown in Table 1.

One can see that adding 6% graphene leads to the 1.5% increase in LOI, and adding 6% DDM-DOPO leads to a 4.1% increase.

All the samples, including the pure sample, passed the UL-94 HB test. Adding the flame retardant reduced the flame spread rates in the UL-94 HB test, and adding DDM-DOPO led to self-extinction of the sample. In the UL-94 HB test, adding graphene and DDM-DOPO led to the decrease in the burning rates of the GFRER samples, and in the VBB test, it led to the decrease in the mass loss rate. According to the LOI and UL-94 HB test data, adding 6% DDM-DOPO more effectively decreased the flammability of the glass fiber-reinforced polymer than adding 6% graphene.

In the VBB test, when permanently exposed to the pilot flame, graphene and DDM-DOPO equally well decreased the mass loss rates. However, the total mass loss proved to be higher for the samples with flame-retardant additives, with the graphene additive raising this parameter higher compared with the DDM-DOPO additive. The increase in the mass loss in the case of adding flame retardants seems to be related to a smaller amount of the char formed, which can be seen from the TGA data. No dripping during VBB burning was found.

### 4.2. Downward Flame Spread over GFRER Composites in Opposed Oxidizer Flow

**Figure 4:**
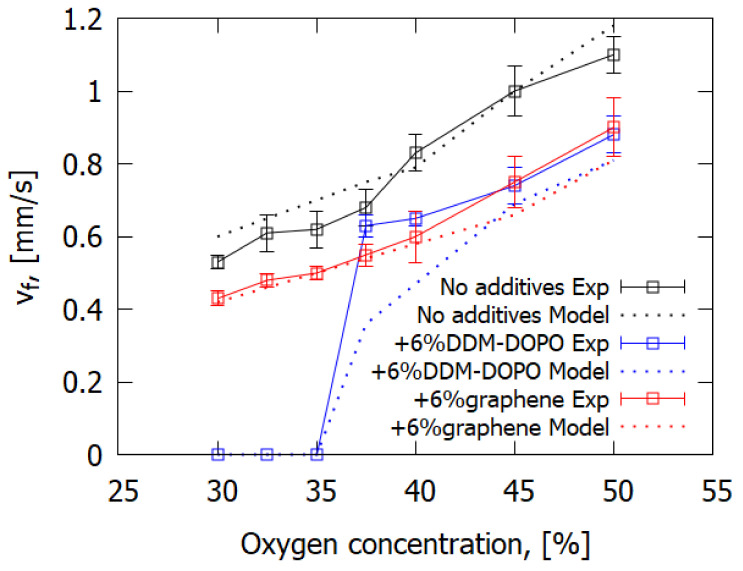
The measured and calculated dependences of ROS on oxygen concentration for downward flame spread over GFRER.

Figure 4 shows the measured and calculated dependencies of the average flame spread rate during downward burning, after the onset of the stationary mode, on oxygen concentration. The increase in oxygen concentration led to the increase in ROS, similarly to the glass fiber-reinforced composites in [18]. Adding 6% graphene or 6% DOPO-DDM led to a reduction in the rate of flame spread. At 35% oxygen concentration, the GFRER + 6% DDM-DOPO samples became self-extinguished in the experiment, whereas for the GFRER and GFRER + 6% graphene samples, self-sustained combustion was observed. DDM-DOPO contains phosphorus; therefore, it is supposed that the degradation products of DDM-DOPO formed in the gas phase participate in the recombination reactions of radicals H and OH, resulting in flame extinction. In the experiment, the effect of both flame retardants regarding the reduction in ROS at O_2_ concentrations greater than 37.5% was practically similar, which agrees with the results of the VBB test.

The modeling data shown in Figure 4 satisfactorily predict a change in the rate of flame spread depending on oxygen concentration both for the pure sample and for the samples with graphene and DDM-DOPO additives. In modeling, self-extinction of the sample with 6% DDM-DOPO additive occurs for 35% O_2_ concentration (limiting oxygen concentration) and lower. In a similar way, self-extinction for this case is observed in the experiment, which is related to insufficient heat release in the gas phase for self-sustained flame spread. The model well predicts the dependence of ROS on O_2_ concentration for samples with the graphene additive, which reduces the amount of the combustible pyrolysis products participating in the reactions of burning and raises the amount of non-combustible pyrolysis products (gaseous soot). At the same time, the model slightly underpredicts the dependence of ROS on O_2_ for GFRER + 6% DDM-DOPO. The volatile phosphorus-containing products of DDM-DOPO pyrolysis reduce the rates of the gas-phase chemical reactions. Variance exceeding the experimental accuracy is observed only at oxygen concentrations less than 45%, which seems to be related to the simplicity of the reaction mechanism used in the model. To be true, using a detailed mechanism of reactions in the gas phase will improve agreement with the experiment; however, the calculation time will significantly increase.

**Figure 5:**
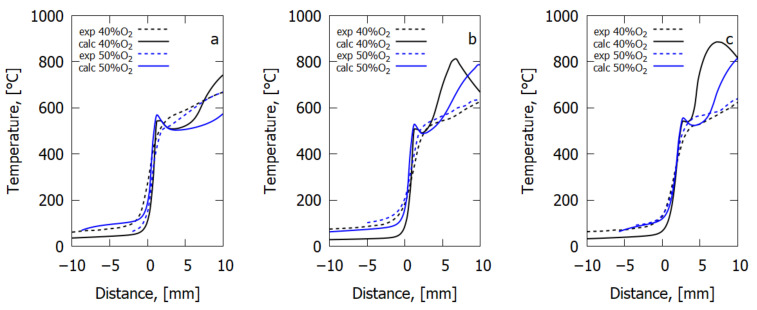
The temperature of the surface slabs (**a**) GFRER, (**b**) GFRER + 6% graphene, (**c**) GFRER + 6% DDM-DOPO. The experiment and the model.

A comparison of the calculated temperature profiles and those measured with the IR camera on the composites’ surface at flame spread is shown in Figure 5. The temperature profiles in the model and in the experiment are close in the preheating area (≈0 mm from the flame front). Due to active soot formation, temperature far from the flame front may be measured by an IR camera with an error, as the surface radiation factor varies. Therefore, comparison of the temperature profiles is shown for the distances from the flame front from −10 to 10 mm. Yet, the major contribution to the rate of flame front shifting (ROS) is made by the temperature gradient from the flame to the sample surface near the flame front, where, as the observations have shown, there is no soot accumulation; therefore, ROS is well predicted by the model (Figure 4).

The characteristic knee of the temperature profile was observed in the experiment near 0 mm at the temperature of 450–500 °C. In the model, a temperature peak was observed near the flame front, whereas in the experiment, it may be missing for two reasons: (1) the low temporal resolution of the IR camera (1 Hz corresponds to approximately ≈1 mm) and (2) the start of soot formation preventing direct observation of the sample surface by the IR camera.

At the distance of 0–10 mm from the flame front (before the start of active soot formation) in the experiment, adding flame retardant did not affect the maximum value of the sample’s surface temperature. The preheating rate in the pyrolysis front (0 mm) did not depend on the presence of the additive and was predicted by the model. Figure S2 (Supplemental Materials) shows distributions of the temperature of the sample surface at downward flame spread, which were obtained with an IR camera. One can see soot formation in the flame front and the fact that as the oxygen concentration grows, its amount on the burning surface increases. In the case of the DDM-DOPO additive, apart from soot on the surface, it is possible to see the formation of phosphorus-containing decomposition products of DDM-DOPO.

**Figure 6:**
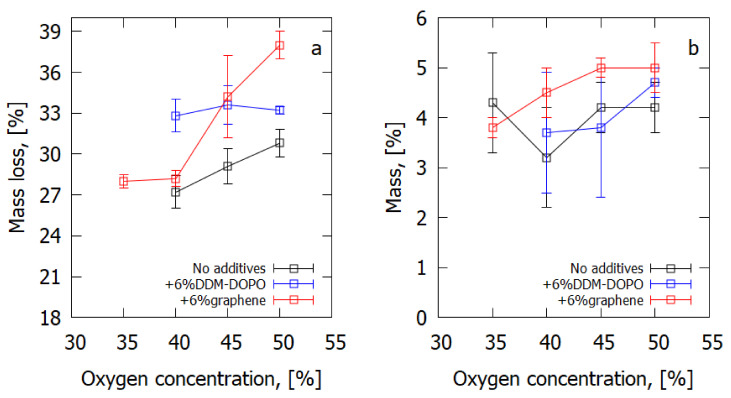
(**a**) Measured total mass loss of a sample after the experiment, and (**b**) the mass of soot collected from the sample surface after the experiment.

Figure 6 shows dependences of the total mass loss and of the mass turned into soot (deposited on the surface) on oxygen concentration in the N_2_/O_2_ flow for GFRER composites. The values provided in Figure 6 are shown in mass percentages from the mass of the original burning part of the sample (20 mm). An increase in oxygen concentration leads to the increase in the amount of formed soot for the GFRER composites with flame-retardant additives. Adding graphene promotes soot formation, especially at higher oxygen concentration. In the case of DDM-DOPO, the measurement error does not allow the increase in the amount of soot on the sample surface to be explicitly determined compared with a sample without additives. At high oxygen concentration (>40% O_2_), greater mass loss is observed in GFRER samples with flame retardants than in pure GFRER samples, which agrees with the thermogravimetric analysis data. This may have a positive effect on the fire protection properties due to the increase in the velocity of the flame retardant’s release onto the surface and in the gas phase. For oxygen concentrations below 35%, no measurements were made due to the difficulty of measuring small (≈20 mg) soot masses.

The addition of DDM-DOPO reduces the flammability of GFRER. The effect of DDM-DOPO in the gas phase is confirmed by the cone calorimeter data in [12] and is consistent with the simulation results. Volatile phosphorus-containing products of DDM-DOPO pyrolysis reduce the overall heat release rate of gas-phase chemical reactions by capturing oxidizing radicals from the origin fuel (here, the gaseous pyrolysis products of epoxy resin), which are key actors in the combustion process. The addition of DDM-DOPO leads to an increase in soot formation in the gas phase.

Thus, it is likely that graphene goes to the surface and serves as a promotor of soot formation. Gaseous products of glass fiber-reinforced composite degradation leave the solid substance, but a certain share of them do not take part in oxidation with oxygen but rather are deposited on the sample surface as soot. It is likely that graphene serves as a condensation site for the resin decomposition products, leading to a smaller amount of the burnt fuel in the gas phase and, accordingly, a smaller heat flux from the flame onto the polymer surface. In its turn, the latter leads to reduction in the rate of flame spread. The paper [43] reports that the addition of expanded graphite to rigid polyurethane foam leads to an increase in the degree of incomplete combustion and promotes smoke formation, which is consistent with the observations presented in our work. In addition, there is evidence in the literature that graphene has adsorbing properties [44].

### 4.3. Upward Flame Spread over GFRER Composites Impacted by the Attached Flame (VBB)

Table 1 contains values of the mass loss rates and total mass loss of GFRER composites at upward combustion when exposed to the flame from an open source, similarly to the vertical Bunsen burner test. The mass loss rate at adding the flame retardants reduced by 33%, and the amount of burnt fuel increased (in the case of adding graphene, more fuel burnt than in the case of adding DDM-DOPO). At upward combustion, more complicated conditions of external heating and the flotation effects are implemented than at downward combustion (photos are presented in Figure S3, Supplemental Materials). The ambivalent results of the VBB test on the total mass loss seem to be related to the complex mechanism of decomposition of resin DGEBA in an oxidative medium [40], as under conditions of upward combustion, the slab is heated at the initial moment of time by a flame source under the sample, with oxidative destruction of the polymer taking place. Yet, the burning velocity, which reduces at adding both flame retardants, is the key parameter which has impact on the flammability of the solid fuel.

**Figure 7:**
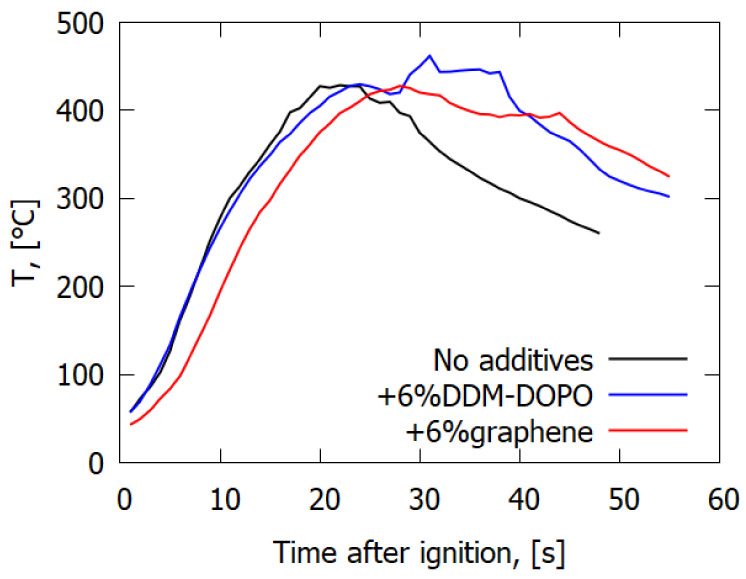
Dependence of the sample surface temperature on time in a VBB test for GFRER.

Dependence of the temperature of the sample surface on time along the centerline of the slab is shown in Figure 7. Whereas the samples without additives and with the 6% DDM-DOPO additive were heated almost simultaneously, in the case of the sample with the graphene additive, a heating delay of 2–3 s was observed. For the pure sample, a peak-form dependence of temperature on time was observed with the maximum at 430 °C. For the samples with additives, two peaks were observed (the second peak took place after passing the flame front and was related to soot formation). The temperatures of the first peak maximum coincided for all the samples. The delay in the heating of the sample surface in the case of adding graphene seems to be related to the fact that when graphene is added, the amount of formed soot, i.e., the amount of incombustible pyrolysis products, increases, resulting in incomplete combustion and, accordingly, in less release of energy into the flame, heating the sample surface.

Thus, DDM-DOPO acts as a flame retardant in the gas phase, while graphene acts in the solid and gas phases.

## 5. Conclusions

For the first time, next to flammability tests (LOI, UL-94 HB, VBB, TGA), experimental studies and the modeling of flame spread and combustion of glass fiber-reinforced epoxy resins with 6% graphene and 6% DDM-DOPO flame-retardant additives have been conducted. The downward flame rates of spread in opposed flow with oxidant and the burning velocity of upward flame over GFRER composites have been measured as well as the distribution of the sample surface temperatures of the composites with graphene and DDM-DOPO flame-retardant additives and without them. Both additives equally decrease the flammability of GFRER. The flammability tests (LOI and UL-94 HB) demonstrate a significant reduction in GFRER flammability upon adding flame retardants. Adding flame retardants results in increased soot formation in the gas phase, and adding graphene results in increased soot deposition on the burning surface. Adding DDM-DOPO results in the increase in the rate of formation of the volatile pyrolysis products, indicating, together with the literature data, the gas-phase mechanism of its action. Adding graphene results in a condensation of the gaseous pyrolysis products on the burning surface, thus reducing the effective amount of fuel participating in the oxidation reactions in the gas phase and indicating the action of the flame retardant in the gas and condensed phase. The developed numerical combustion model for GFRER with DDM-DOPO additive, based on the action of DDM-DOPO as a flame retardant acting in the gas phase, has demonstrated satisfactory accuracy in predicting reduction in downward ROS over GFRER. The developed numerical combustion model for GFRER with the graphene additive, based on the reduction in the amount of combustible fuel and increase in the amount of non-combustible volatile pyrolysis products when graphene is added, has demonstrated good accuracy in predicting downward ROS over GFRER. The data obtained may be used for designing effective reinforced non-combustible composites applied in the aircraft industry and for determining a detailed mechanism of the effect of flame-retardant additives.

## Figures and Tables

**Table 1:** The LOI, UL-94, and TGA data, the mean mass loss rate (MLR), and the total mass loss (TML) in the VBB test.

Sample	LOI, %	UL-94 HB with ROS, mm/min	MLR in VBB, g/s	TML in VBB, g	T_max_ DTG in Inert, °C
GFRER	22.4	44.5, burned out ^1^	0.015 ± 0.002	0.51 ± 0.11	412
GFRER + 6% graphene	23.9	38.3, burned out ^1^	0.010 ± 0.002	0.93 ± 0.18	411
GFRER + 6% DDM-DOPO	26.5	34.9, self-extinguished ^1^	0.010 ± 0.002	0.66 ± 0.14	407

^1^ HB rating.

## Data Availability

Not applicable.

## Supplementary Materials

The following supporting information can be downloaded at: https://www.mdpi.com/article/10.3390/polym14163379/s1, Figure S1: Micrographs of the GFRER + 6% graphene surface; Figure S2: The surface temperature distributions during stationary combustion, obtained using an IR camera, for samples at 35%, 40% and 45% O_2_ concentrations; Figure S3: Flame photos of GFRER composites in the VBB test.

## Abbreviations

ADC	analog-to-digital converter
GFRER	Glass fiber-reinforced epoxy resin
LOI	limiting oxygen index
TG	thermogravimetry
DTG	differential thermogravimetric analysis
**Nomenclature**	
*C*	specific heat capacity (J/kg/K)
D	diffusion coefficient (m^2^/s)
E	activation energy (J/mol)
g	gravity acceleration (m/s^2^)
k	preexponential factor (s^−1^)
M	molar mass (g/mol)
$\dot{m}$	mass burning rate (g/s)
n	pyrolysis reaction order (−)
p	pressure (Pa)
Q	heat release (J/kg)
q	heat flux (W/m^2^)
$R_{0}$	universal gas constant (J/mol/K)
T	temperature (K)
t	time (s)
u	velocity (m/s)
W	reaction rate (s^−1^)
x	coordinate along fuel surface (m)
Y	gas component mass fraction (−)
y	coordinate normal to fuel surface (m)
**Greek symbols**	
α	conversion degree (−)
γ	solid component mass fraction (−)
δ	burnout degree (−)
η	solid component volume fraction (−)
λ	thermal conductivity (W/m/K)
μ	dynamic molecular viscosity (kg/m/s)
ν	stoichiometric coefficient (−)
ρ	density (kg/m^3^)
τ	half-thickness (m)
ψ	inhibition effect coefficient
**Subscripts**	
a	ambient
b	binder
F	fuel
f	fiber
g	gas
I	inert component
O	oxidizer
P	product
p	pyrolysis
s	solid
**Superscripts**	
r	radiative
x	along fuel surface
y	normal to fuel surface
