# Supplementary material for: The Influence of Flame Retardants on Combustion of Glass Fiber-Reinforced Epoxy Resin
Source: Polymers (Basel). 2022 Aug 18;14(16):3379. doi: 10.3390/polym14163379 (PMC9416137; doi:10.3390/polym14163379)
Supplement: Supplementary file 1 [file polymers-14-03379-s001.zip › polymers-1863570-supplementary.pdf]

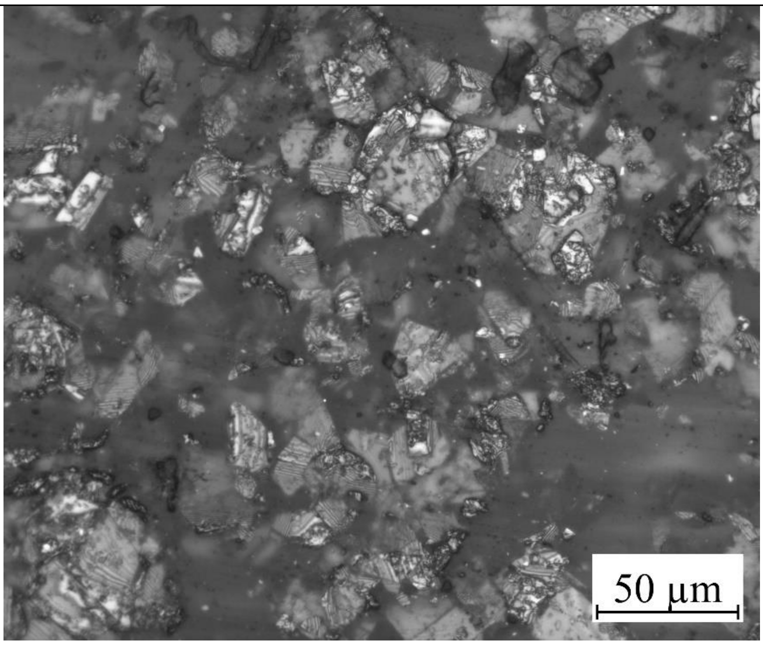


Optical microscopy

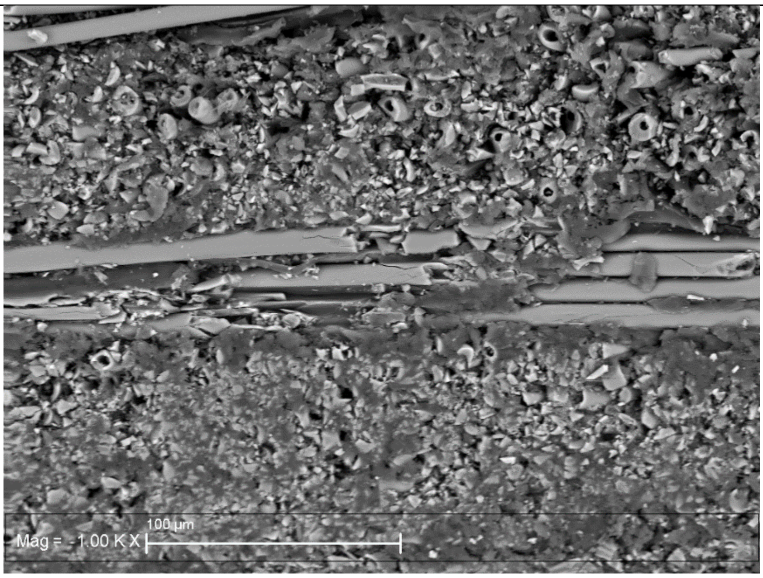


SEM microscopy

Figure S1. Micrographs of the GFRER+6%graphene surface.

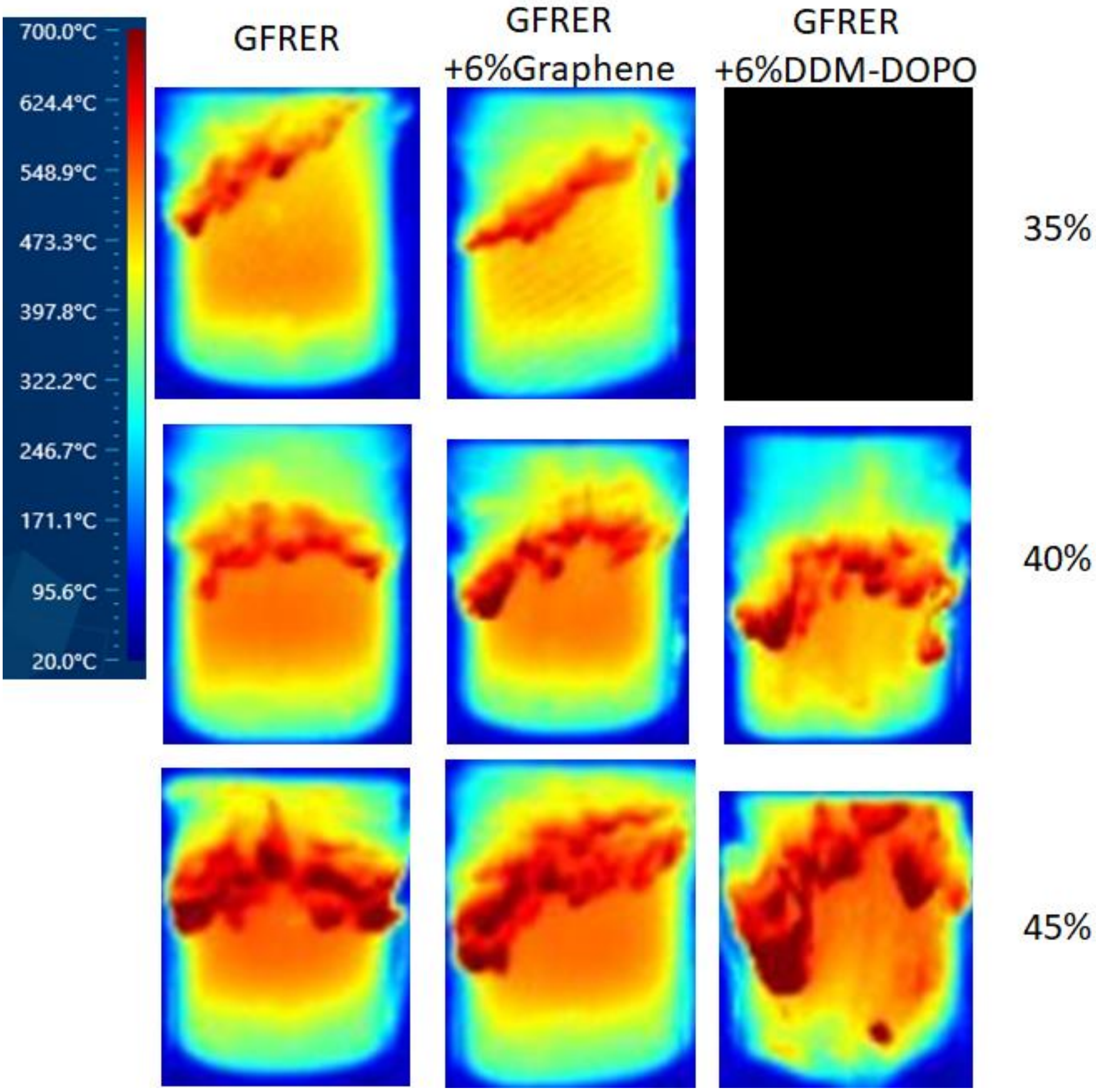


**Figure S2.** The surface temperature distributions during stationary combustion, obtained using an IR camera, for samples at 35%, 40% and 45% $O_2$ concentrations.

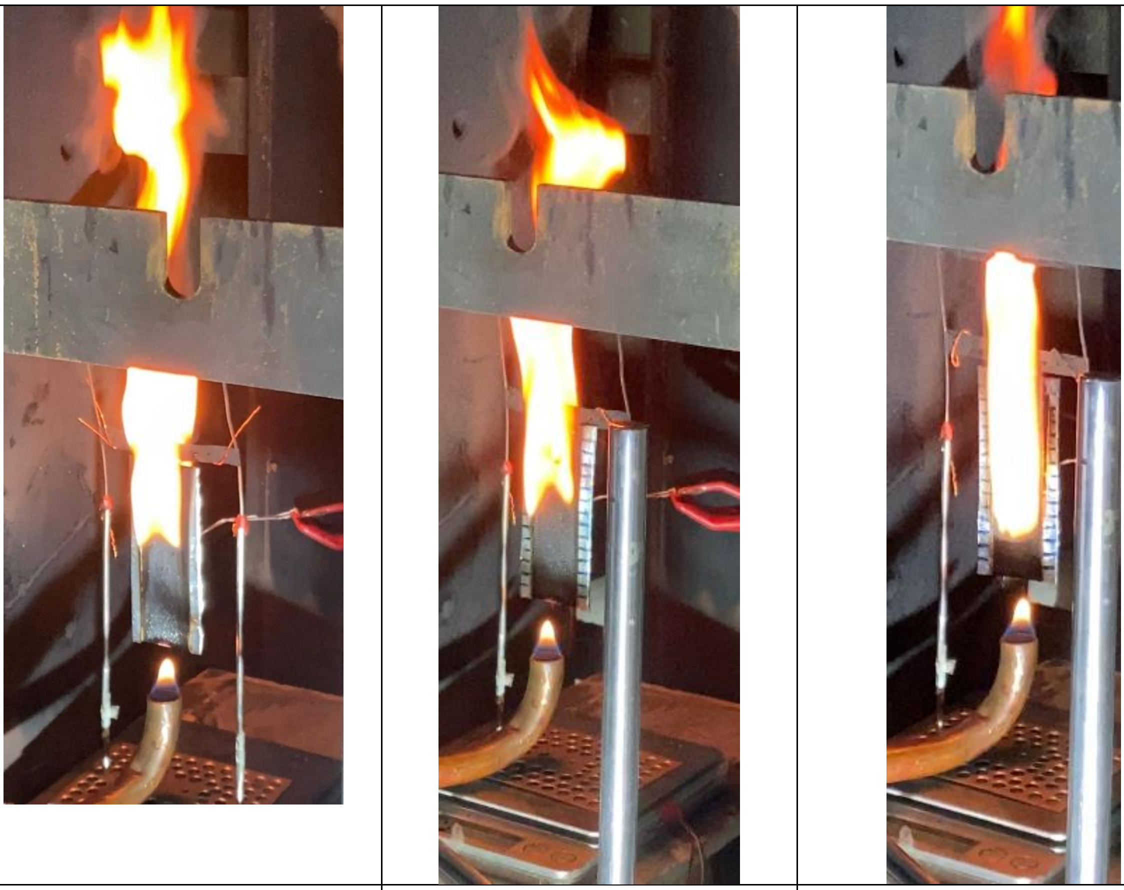

| GFRER | GFRER+6%graphene | GFRER+6%DDM-DOPO |
|---|---|---|

**Figure S3.** Flame photos of GFRER composites in the VBB test.
